# ﻿Chromosomal polymorphism in natural populations of *Chironomusborokensis* Kerkis, Filippova, Shobanov, Gunderina et Kiknadze, 1988 (Diptera, Chironomidae)

**DOI:** 10.3897/compcytogen.19.141735

**Published:** 2025-04-15

**Authors:** Veronika V. Golygina, Andrey D. Broshkov, Oksana V. Ermolaeva

**Affiliations:** 1 The Federal Research Center Institute of Cytology and Genetics of Siberian Branch of the Russian Academy of Science, Prospect academika Lavrentieva 10, Novosibirsk 630090, Russia The Federal Research Center Institute of Cytology and Genetics of Siberian Branch of the Russian Academy of Science Novosibirsk Russia; 2 Novosibirsk State University, ul. Pirogova, 2, Novosibirsk 630090, Russia Novosibirsk State University Novosibirsk Russia

**Keywords:** Banding sequence, B-chromosome, *Ch.plumosus* group, inversion, karyological analysis, karyotype, polytene chromosome, sibling species, translocation

## Abstract

Chromosomal polymorphism has been studied in 31 natural populations of *Ch.borokensis*. For 11 out of 31 populations quantitative analysis of chromosomal polymorphism has been performed. Data from previous publications (10 populations) have also been used to perform an overview of the chromosomal polymorphism of *Ch.borokensis* and to establish the species range. Most studied populations show a high level of chromosomal polymorphism: on average 66.1 ± 2.6% of specimens were heterozygotes with 1.1 ± 0.6 heterozygotic inversions per larvae. The number of banding sequences found in populations varied from 7 to 18 per population with an average of 12 ± 3. Inversions were found in all chromosomal arms. Besides inversions, B-chromosome was present in 13 populations, and 5 translocations were found. The total number of banding sequences found in banding sequence pool of *Ch.borokensis* is 40. Twenty six banding sequences are described for the first time for the species.

## ﻿Introduction

The species *Chironomusborokensis* Kerkis, Filippova, Shobanov, Gunderina et Kiknadze, 1988 is one of the 14 sibling species in the *Ch.plumosus* group. The group presents a good model for studies of evolution of karyotype during speciation as well as chromosomal polymorphism in natural populations and its correlation with environmental conditions. Three species with widest ranges – *Ch.plumosus* (Linnaeus 1758), *Ch.entis* Schobanov, 1989 and *Ch.balatonicus* Dévai, Wülker et Scholl, 1983 – had been studied most extensively (Kiknadze et al. 2016). At the same time, only few populations were studied karyologically for other species from the group. In case of *Ch.borokensis* data on chromosomal polymorphism were published for several populations from Europe, northern and central part of the Ural, Eastern Siberia and the Far East (Kerkis et al. 1988; Golygina et al. 2003; Petrova and Klishko 2005; Proviz and Bazova 2013; Filinkova 2019). These studies had shown that the species has considerable level of chromosomal polymorphism in regards to number of heterozygotes in populations, but in total only 7 inversions besides main banding sequences were found in all studied populations, so the total number of banding sequences known until now for *Ch.borokensis* was 14. Five of those inversions – h’borA2, p’borB2, h’borD2, p’borF2 and p’borG2 can be classified as alternative banding sequences of the species as all of them were found both in hetero- and homozygote state in several populations. Yet almost nothing were known about polymorphism in populations from Western Siberia – only 5 larvae from Novosibirsk were studied in the work of Kerkis et al. (1988) – despite the fact that this region probably represents the central part of the species range judging from its previous findings both to the east and to the west of it. Thus, in order to understand the true level of chromosomal polymorphism of *Ch.borokensis* it was very important to obtain data from Western Siberia.

In this study we present our data on chromosomal polymorphism in 31 natural populations of *Ch.borokensis* from Eastern Europe, Western Siberia and the Far East, and also include data for 10 populations published by other authors (Kerkis et al. 1988; Petrova and Klishko 2005; Proviz and Bazova 2013; Filinkova 2019) in order to perform comprehensive analysis of patterns of chromosomal polymorphism of this species.

## ﻿Material and methods

Fourth instar larvae were used for polytene chromosome slide preparations from 29 natural populations of Eastern Europe, Siberia and the Far East. For 2 populations from the Far East (Khabarovsk krai) and 2 samples collected in 1985 in Berdsky Pond (Novosibirsk province) permanent slides housed in the collection of the Institute of Cytology and Genetics SB RAS (Novosibirsk, Russia) were used for repeat analysis of chromosomal polymorphism as original data published by Kerkis and coauthors (Kerkis et al. 1988), did not contain comprehensive data suitable for quantitative analysis. Data on collection sites are presented in Table 1. In cases when larvae were obtained several times over the years from the same collection site statistical analysis (Fisher criteria, Plokhinsky 1967) was performed to determine if there is a significant difference in sequence frequencies between samples from different years. If no differences were found data on all probes were combined. Data for 10 populations published previously (Kerkis et al. 1988; Petrova and Klishko 2005; Proviz and Bazova 2013; Filinkova 2019) were also used for analysis of chromosomal polymorphism (Table 2), in cases if frequencies of banding sequences were not present in publications they were calculated from the data on frequencies of genotypic combinations.

**Table 1.** Collection sites.

Collection locality	Abbreviation	Collection date	Geographic coordinates	Number of larvae
**RUSSIA**				
**Sankt-Petersburg**				
Pond in Sankt-Petersburg city	LEN-P1	18.02.1987	59°39'43"N, 30°14'58"E	1
**Yaroslavl province**				
Barsky Pond, Borok settlement	YAR-BA	15.12.1990	58°03'42"N, 38°14'45"E	7
Rybinsk Reservoir	YAR-RY	09.09.1988	58°11'59"N, 38°24'59"E	1
**Nizhny Novgorod province**				
Lake Yurievskoe, Yurievets settlement, Dzerzhinsk city district	NNO-YU	06.1990	56°12'42"N, 43°37'52"E	1
**Tomsk province**				
Lake Peschanoe, Timiriazevskoe village, Tomsk city district	TOM-PE	08.04.2005	56°27'33"N, 84°52'54"E	1
**Novosibirsk province**				
Bol’shaya Protoka Lake, Rechport, Novosibirsk city	NSK-2R	25.12.1996	54°56'07"N, 83°03'46"E	2
		13.05.2005		1
Berdsky Pond on the Shadrikha Rivulet, Berdsk town	NSK-BE	08.01.1985^†^	54°43'56"N, 83°07'49"E	1
		19.09.1985^†^		2
		7.1988		2
		16.01.1989		5
		03.06.1996		1
		25.06.2007		212
		05.05.2008		93
		23.06.2011		42
		11.05.2012		21
		06.05.2016		5
		15.06.2021		84
		14–28.06.2023		242
		29.06.2024		24
Kainka Lake, Kainskaya Zaimka settlement	NSK-KA	05.05.1997	54°52'14"N, 83°08'10"E	1
		10.05.2001		1
Pond on the Chernodyrikha River, near	NSK-CH	16.05.2006	54°35'60"N, 83°07'58"E	16
Ryabchinka village				
Pond on the Ora River, Smolensky settlement	NSK-OR	17.05.2002	55°12'05"N, 83°20'46"E	20
		16.05.2005		46
		12.05.2006		68
Pond on the Sarboyan River, Uchastok-Balta village	NSK-SA	16.05.2002	55°25'29"N, 83°56'14"E	5
Pond on the Kaily River, Yoltishevo village	NSK-KY	16.05.2002	55°21'30"N, 84°05'29"E	34
Pond on the Tars’ma River, Stepnogutovo settlement	NSK-ST	12.05.2011	54°51'09"N, 84°57'32"E	5
**Altai Krai**				
Travyanoe Lake, Oskolkovo village	ALT-TR	08.05.1994	52°19'12"N, 83°11'24"E	9
Anisimovo Lake, Cheryomnoe village	ALT-AN	15.02.2006	53°10'26"N, 83°14'58"E	116
Pond on the Salandaika River, near Lugovoe village	ALT-SA	06.05.1997	52°20'32"N, 86°09'31"E	117
**Republik of Altai**				
Manzherok Lake, near Manzherok village	RAL-MA	07.05.1997	51°49'15"N, 85°48'50"E	123
**Kemerovo province**				
Tanaevo Lake, Zhuravlevo village	KEM-TA	14.05.2002	54°46'35"N, 85°02'52"E	3
Pond near Krasninskoe village	KEM-KR	15.05.2004	54°47'29"N, 85°26'00"E	1
Pond in Anzhero-Sudzhensk town	KEM-AN	21.05.2004	56°04'57"N, 86°01'23"E	2
Old channel of Yaya River, near Yaya town	KEM-YA	20.05.2004	56°12'42"N, 86°29'37"E	1
Big pond on the Suluyul River, Krasnie Orly village	KEM-SU	19.05.2004	56°09'33"N, 87°13'51"E	100
Pond on the Baim River, Novokazanka village	KEM-BA	16.05.2004	56°06'38"N, 87°35'37"E	94
Small Pond near Oboyanovka village	KEM-OB	18.05.2004	56°21'04"N, 87°36'20"E	8
Old channel of Kiya River	KEM-KI	17.05.2004	56°09'15"N, 87°44'28"E	2
**Irkutsk province**				
Kuz’mihinskoe Lake, Irkutsk city	IRK-KU	15.04.2006	52°14'34"N, 104°17'38"E	1
**Republik Saha-Yakutia**				
Saisari Lake, Yakutsk city	YAK-SA	10.1998	62°00'52"N, 129°41'36"E	6
**Khabarovsk Krai**				
Udil Lake^†^	KHA-UD	21.06.1987	52°08'34"N, 139°58'46"E	2
Chlya Lake^†^	KHA-CH	06.1987	53°22'49"N, 140°01'24"E	17
**Primorsky Krai**				
Tumannaya River, near Khasan settlement^‡^	PRI-TU	25.05.1998	42°24'53"N, 130°38'37"E	7
Chan Lake, Vladivostok city^‡^	PRI-CH	07.05.1999	43°08'32"N, 131°54'23"E	17

^†^ - permanent slides presented in collection stored in the Institute of Cytology and Genetics SB RAS (Novosibirsk, Russia) were used for repeat analysis of this population, original data was published by Kerkis and coauthors (Kerkis et al. 1988), but without comprehensive quantitative data. ^‡^ - data obtained by authors were earlier published in the paper describing new species *Ch.suwai* (Golygina et al. 2003) for the purpose of comparison of its inversion polymorphism.

**Table 2.** Populations from published papers of other authors.

Collection locality	Abbreviation	Collection date	Geographic coordinates	Number of larvae	Source
**RUSSIA**					
**Yaroslavl province**					
Pond in Grigorovo village near Borok settlement	YAR-GR	04.04.1987	58°04'08.1"N, 38°13'31.7"E	100	Kerkis et al. 1988
**Sverdlovsk province**					
Old channel of Lozva River ‘Osinovskaya’, Ivdelya town	SVE-OS	28.09.2008	Not presented	30	Filinkova 2019
Karst hollow near Kalya settlement, Severouralsk city district	SVE-KH	08.08.2009	Not presented	45	Filinkova 2019
Temporary waterbody near Boksiti settlement	SVE-BO	05.11.2009	Not presented	43	Filinkova 2019
Yukonka pond, 42 km from Verhoturye town	SVE-YU	01.04.2007	Not presented	16	Filinkova 2019
Pond in open pit, 40 km from Verhoturye town	SVE-VP	03.05.2008	Not presented	12	Filinkova 2019
Kryakva Pond, 38 km from Verhoturye town	SVE-KR	03.05.2008	Not presented	29	Filinkova 2019
**Irkutsk province**					
Kotokel Lake	IRK-KO	15.06.2009	52°48'16"N, 108°07'59"E	26	Proviz and Bazova 2013
Dukhovoe Lake	IRK-DU	26.05.1983	53°17'11"N, 108°51'49"E	30	Proviz and Bazova 2013
**Zabaikalsky Krai**					
Ivan Lake	ZAB-IV	04.1997	52°14'36"N, 112°58'40"E	10	Petrova and Klishko 2005

Species identification was done by analysis of larval morphology and polytene chromosomes from the salivary glands. The stage of larval development was determined based on the shape of the imaginal disks in the first two thoracic segments. The larvae were fixed with 3:1 *v/v* of 96% ethanol and glacial acetic acid and stored at – 20 °C. Polytene chromosome squashes were prepared by the routine aceto-orcein method (Keyl and Keyl 1959; Kiknadze et al. 1991). Chromosomal mapping of arms A, C, D, E and F was done using mapping system created by Keyl (1962) and Dévai et al. (1989), with *Ch.piger* Strenzke, 1959 as the standard karyotype. Mapping of arms B and G was done according to Maximova (1976) mapping systems improved by Shobanov (1994a), with *Ch.plumosus* chromosomes as the standard. In cases of pericentric inversions bands that originated in other arm was prefixed with the arm designation, i.e. if, for example, region 15c-12v of the arm B was transferred into the arm A, it was shown in mapping of the arm A as B15c-B12v.

Each banding sequence is given a short designation as follows: three-letter abbreviation of the species name (bor for *Ch.borokensis*) followed by the name of the arm and the serial number of banding sequence in this arm (according to the order of its discovery), and prefixed by a letter indicating its geographical distribution in the genus *Chironomus* (p’ for Palearctic sequences or h’ for Holarctic sequences). Thus, for example, h’borE1 means that while *Ch.borokensis* itself is a Palearctic species, this banding sequence is identical to banding sequences of some other species and was found not only in Palearctic but also in Nearctic, i.e. this banding sequence has Holarctic distribution.

All banding sequences were classified into four classes according to the system suggested by Gunderina et al. (1999a) and Golygina (1999): main banding sequences (present in most populations of a species both in homo- and heterozygotic state, dominant in most populations of a species), alternative banding sequences (present in many populations of a species both in homo- and heterozygotic state, but their frequencies exceed frequencies of main banding sequences only in some populations), rare banding sequences (found with low frequencies in some populations, can be present in all species range or just in some geographic regions), unique banding sequences (found only once in one population).

For analysis of origin of an inversion banding sequence the term “hypothetical” banding sequence is used for intermediate banding sequences in cases where there are several inversion steps between existing banding sequences but no intermediate banding sequences were found in studied populations. Number of inversion steps is determined based on the number of breakpoints that separate two compared banding sequences.

Cytogenetic structure of populations was designated in accordance with the system, suggested by Gunderina et al. (1999a). Frequencies of main banding sequences were used for determining of the type of populations: type 0 – if all main banding sequences are dominant (frequencies higher than 0.5), type B – if frequency of main banding sequence in arm B is below 0.5, type CD – if frequency of main banding sequence in arms C and D are below 0.5 etc.

All populations were checked for deviations from Hardy-Weinberg equation.

Statistical analysis and phylogenetic tree construction was done using programs Excel 2013 (Microsoft Office 2013), PHYLIP (https://phylipweb.github.io/phylip/https://evolution.genetics.washington.edu/phylip.html) and MEGA11.0.8 (https://www.megasoftware.net).

The equipment of the Centre for Microscopic Analysis of Biological Objects (Institute of Cytology and Genetics SB RAS, Novosibirsk, Russian Federation) was used for this work: microscope “Axioskop” 2 Plus, CCD-camera AxioCam HRc, software packages AxioVision 4 and Zen (Zeiss, Germany).

## ﻿Results and discussion

**Figure 1.**
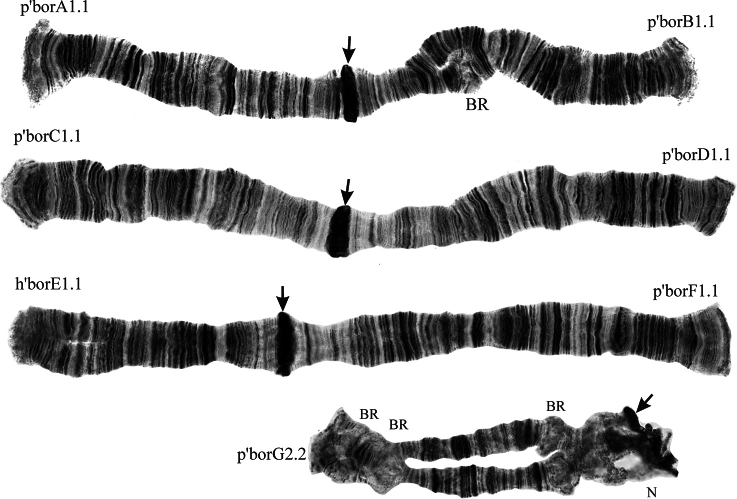
Karyotype of *Chironomusborokensis* (the authors’ photo was originally published in Kiknadze et al. 2016); p’borA1.1, p’borB1.1 etc. – genotypic combinations of banding sequences; BR – Balbiany Rings, N – nucleolus; arrows indicate centromeric bands.

**Figure 2.**
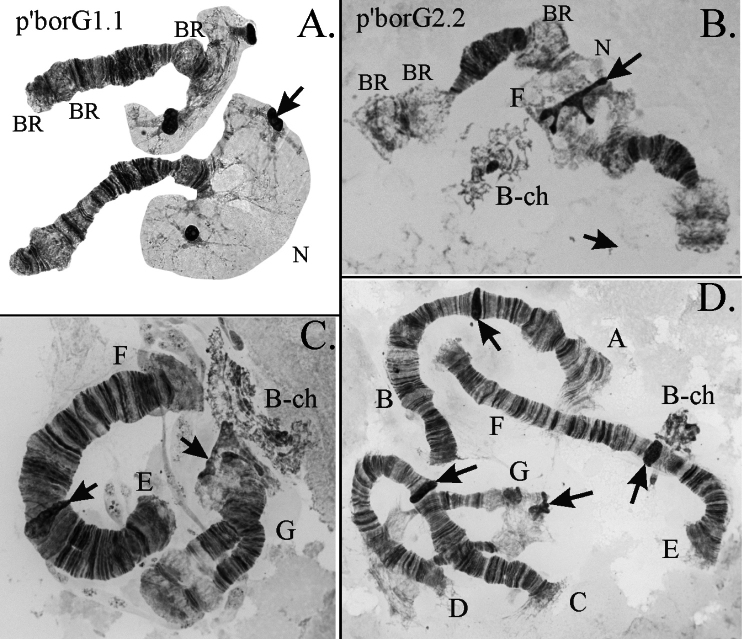
Main banding sequence of arm G (**A** the authors’ photo was originally published in Kiknadze et al. 2016) and B-chromosome (**B–D**) of *Ch.borokensis*; p’borG1.1 etc. – genotypic combinations of banding sequences; letters A, B etc. – chromosomal arms, BR – Balbiany Ring, N – nucleolus, B-ch – B-chromosome, arrows indicate centromeric bands.

*Ch.borokensis* belongs to “thummi” cytocomplex with haploid number of chromosomes n=4 and arm combination AB CD EF G. The chromosomes I (AB) and II (CD) are metacentric, III (EF) is submetacentric, and IV (G) is telocentric (Fig. 1). Homologues of arm G are often unpaired or conjugated only at the end in the nucleolus region. Centromeric regions are very large and easily identifiable on all chromosomes. There is one nucleolus in *Ch.borokensis* karyotype, that is situated on the arm G under the centromere. There are four Balbiani Rings (BR): three are situated on the arm G (one under the nucleolus and two on the opposite end near telomere (Figs 1, 2), the activity of them can vary so not all three BR are always visible), and the fourth one – on the arm B. Unlike in many other species from the genus *Chironomus*, BR on the arm B often shows low activity on one or both homologues and in some cases can be completely inactive (Fig. 1).

**Figure 3.**
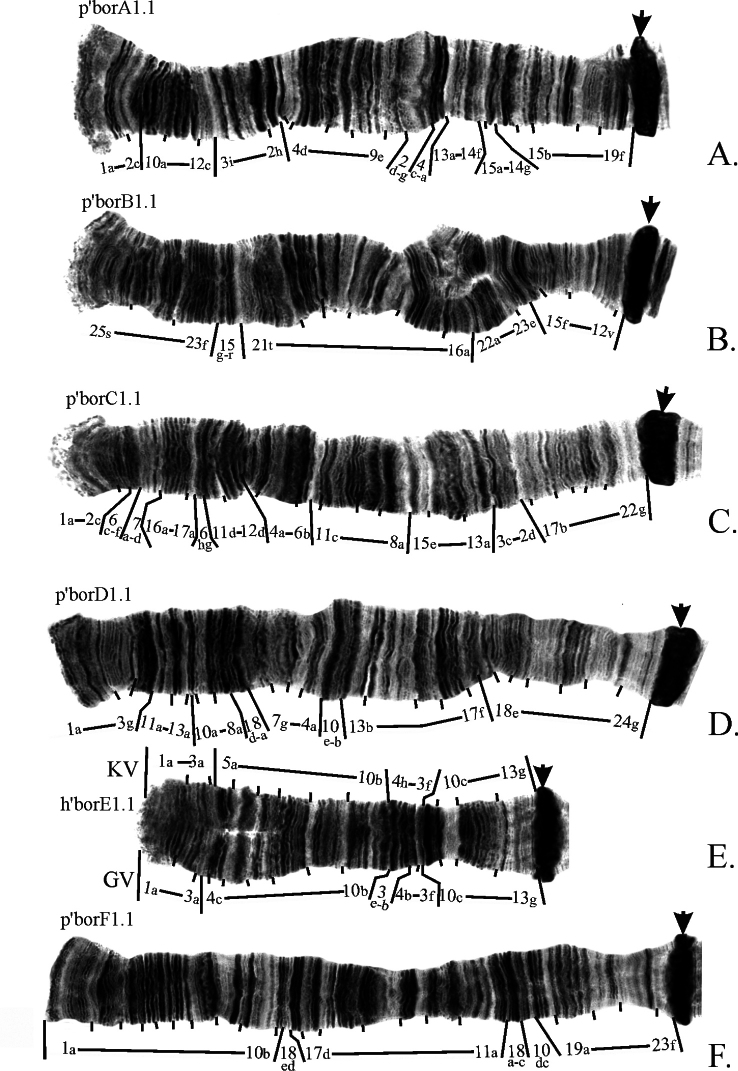
Mapping of main banding sequences in arms **A–F** of *Ch.borokensis* (the authors’ photo was originally published in Golygina and Kiknadze 2008 (**A, B**), Golygina and Kiknadze 2012 (**C, D**) and Kiknadze et al. 2016 (**E, F**)). Arrows indicate centromeric regions. KV – version of mapping in arm E according to Keyl (1962), GV – version of mapping in arm E according to Golygina and Kiknadze (2018).

The revision of mapping of the main banding sequences in arms A, B, C, D, E and F was presented by Golygina and Kiknadze previously (2008, 2012, 2018). Revised mapping of these banding sequences is shown on Fig. 3. For arm E two versions of mapping are presented (Fig. 3E). First is done according to how *Ch.borokensis* banding sequence should be mapped if mapping of *Ch.plumosus* – reference species for mapping of all *Ch.plumosus* group sibling species – made by Keyl (1962) is considered to be correct (marked as KV). The second one is done according to revised mapping of *Ch.plumosus* made by Golygina and Kiknadze (2018) (marked as GV).

Inversion polymorphism was found in all chromosomal arms. Mapping of all banding sequences is presented in Suppl. material 1. Frequencies of occurrence of genotypic combinations of banding sequences are presented in Suppl. material 2, and frequencies of occurrence of banding sequences are presented in Suppl. material 3.

**Figure 4.**
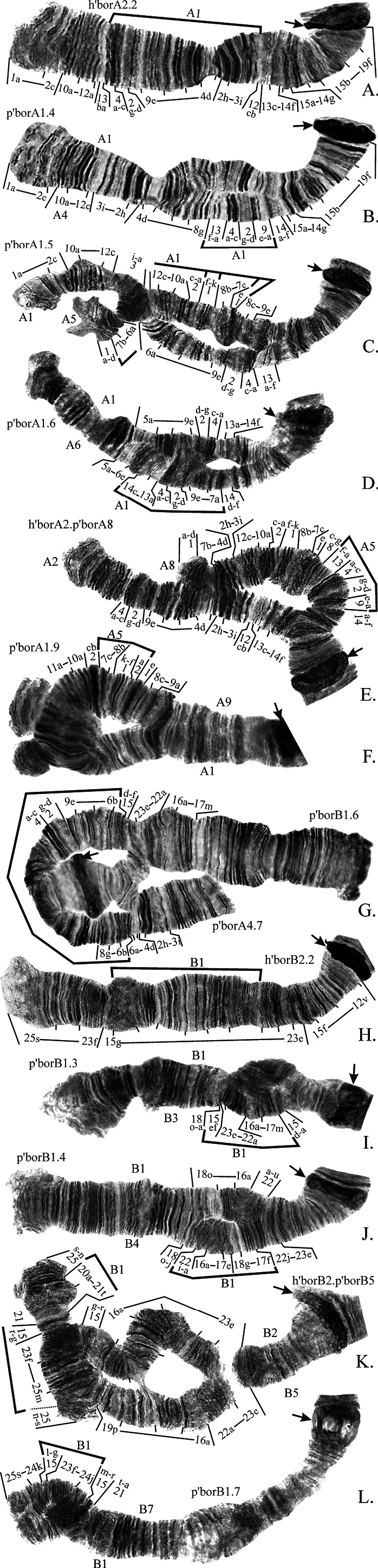
Inversions in chromosome I (arms **A, B**) found in populations of *Ch.borokensis* (photo of h’borB2.2 (N) was made by the authors but was originally published in Kiknadze et al. 2016). Arrows indicate centromeric regions. Brackets show regions of inversions. Regions of chromosomal arms designated by Arabic numerals, small letters indicate separate bands in a region. h’borA2.2, p’borA4.4 etc. – designations of genotypic combination of banding sequences. A1, A2 etc. – shortened designation of banding sequence (i.e. A1 for p’borA1), if placed near chromosome it indicates banding sequence of the particular homologue, if placed above or below a bracket it indicates banding sequence from which the homologue derived from.

**Arm A** has 9 banding sequences (Fig. 4A–G) and was polymorphic in most studied populations. Banding sequences h’borA2 and p’borA4 were found both in hetero- and homozygotic state (Fig. 4A, B, E), the rest occurred only as heterozygotes in combinations with p’borA1 (mostly), h’borA2 or p’borA4 (Fig. 4C–G). Banding sequences p’borA2, p’borA3, p’borA4, and p’borA6 are simple paracentric inversions originated from p’borA1 (Fig. 4A, B, D). The banding sequence p’borA5 is a complex inversion that differs from p’borA1 by three inversion steps, intermediate inversions were not found in any populations so they are designated here as “hypothetical” (Fig. 4C, Suppl. material 4). Banding sequences p’borA8 and p’borA9 appear as complex inversions in comparison to p’borA1, but both are actually simple inversions originated from p’borA5 (Fig. 4E, F, Suppl. material 4).

It is necessary to say that p’borA5, p’borA8 and p’borA9 are very hard to distinguish from one another as the distal part of the arm is identical in all of them and they differ only by relatively short inversions in the proximal part of the arm where the banding structure is often not very clear. The most interesting thing about these group of inversions is that p’borA5 is more ancient than the species *Ch.borokensis* itself. This conclusion can be drawn from the fact that identical banding sequence was also found in sibling species *Ch.suwai* (p’suwA2, Golygina et al. 2003) and, although it was not found in the banding sequence pool of *Ch.plumosus*, two inversions of this species – p’pluA3 and p’pluA7 – can be derived from it by simple inversions (Golygina and Kiknadze 2001). As it is extremely unlikely that identical complex inversion emerged independently in three species, it is safe to assume that the banding sequence p’borA5 should have been already present in the ancestral species that later gave birth to three sibling species – *Ch.plumosus*, *Ch.borokensis* and *Ch.suwai*.

Banding sequences p’pluA3 and p’pluA7 were mapped somewhat differently before (Golygina and Kiknadze 2001), as both are complex in comparison to p’pluA1 and to each other, often have unclear banding structure and so were very difficult to map with certainty. However, the finding of p’borA5 allowed us to better understand the relationships in this cluster of inversions and revise their mapping (Suppl. material 4).

Finally, the banding sequence p’borA7 is a result of the simple pericentric inversion (Fig. 4G). As this inversion affected only short part of the arm B but more than half of the arm A, the inverted homolog of chromosome I (AB) became submetacentric.

**Arm B** has 7 banding sequences (Fig. 4G–L) and was polymorphic in most studied populations. From six inversions only h’borB2 was found both as hetero- and homozygote (Fig. 4H, K). Banding sequences p’borB3, p’borB4, p’borB5 and p’borB7 are paracentric inversions originated from p’borB1 (Fig. 4I–L), and p’borB6 is a result of simple pericentric inversion described above (Fig. 4G).

**Figure 5.**
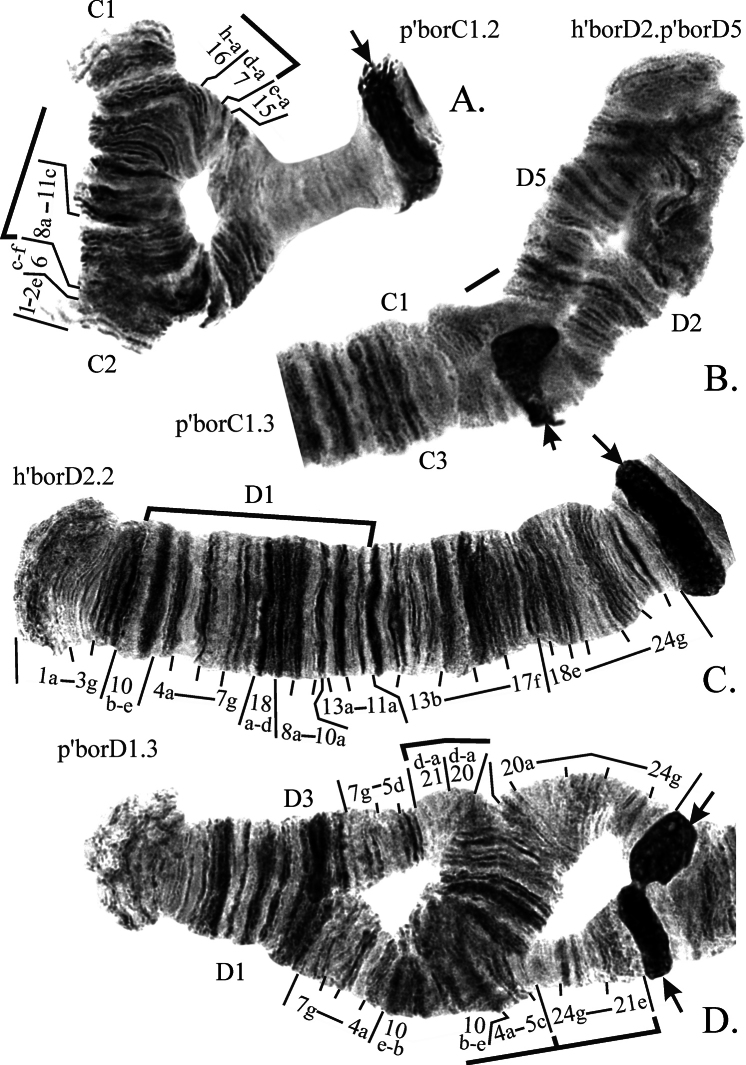
Inversions in chromosome II (arms **C, D**) found in populations of *Ch.borokensis* (photo of h’borD2.2 (**C**) was made by the authors but was originally published in Kiknadze et al. 2016). Designations as on Fig. 4.

**Arm C** has 3 banding sequences (Fig. 5A, B) but overall it was basically monomorphic. The banding sequence p’borC2 is a large simple inversion (Fig. 5A), and p’borC3 is a short pericentric inversion (Fig. 5B).

**Arm D** has 5 banding sequences (Fig. 5B–D, 7C) and was polymorphic in most studied populations. Banding sequences h’borD2 (Fig. 5C) and p’borD3 (Fig. 5D) are simple paracentric inversions, but only h’borD2 was found in hetero- and homozygotic state. The banding sequence p’borD4 is a result of reciprocal translocation (Fig. 7C) between chromosome 2 (CD) and 3 (EF), and p’borD5 is a simple pericentric inversion (Fig. 5B).

**Figure 6.**
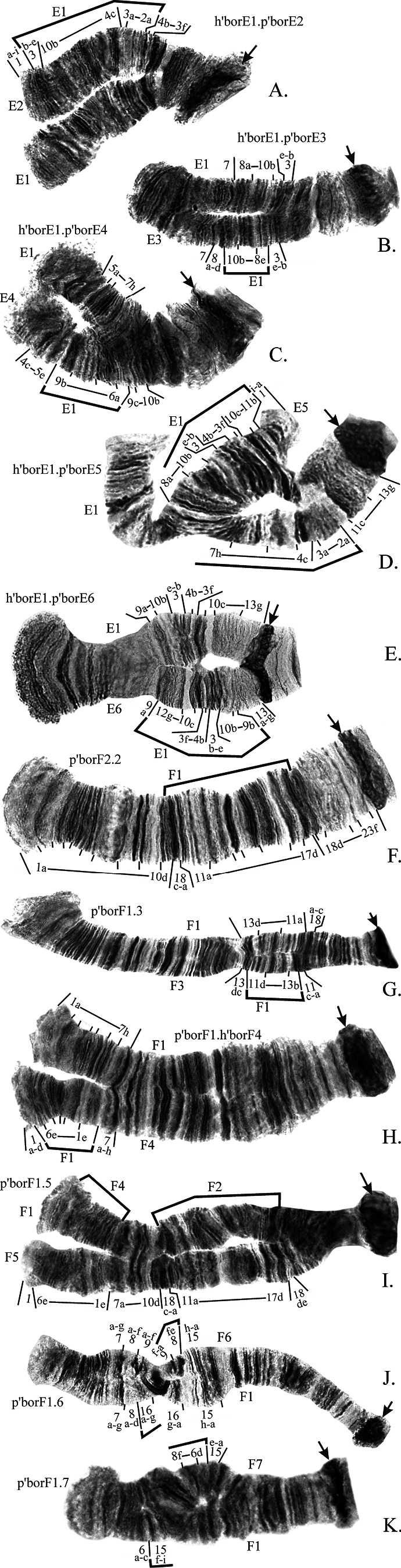
Inversions in chromosome III (arms **E, F**) found in populations of *Ch.borokensis*. Designations as on Fig. 4.

**Figure 7.**
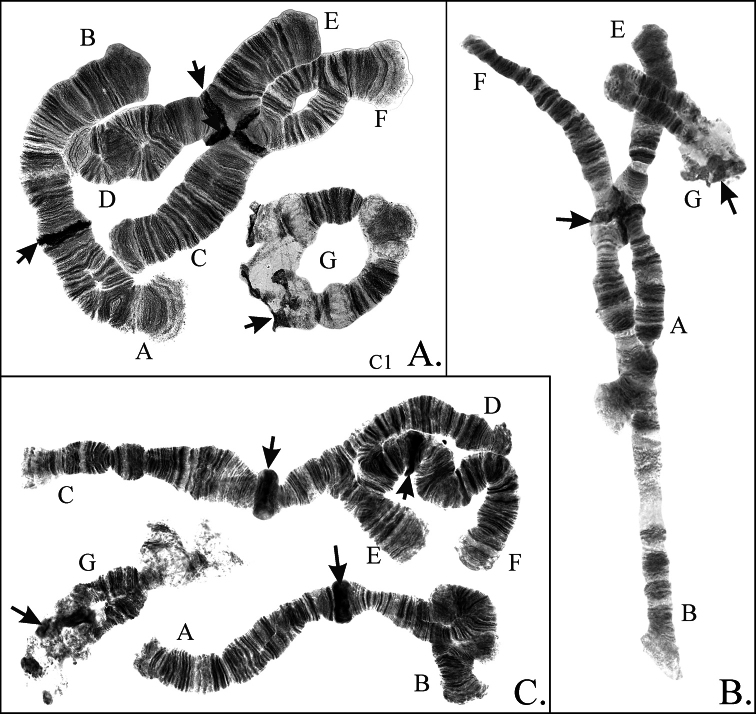
Translocations found in populations of *Ch.borokensis*. Letters A, B, C etc. designate chromosomal arms, arrows indicate centromeric regions.

**Arm E** has 7 banding sequences (Fig. 6A–E, 7C), but, as the arm C, was monomorphic in most studied populations. All inverted banding sequences originated from h’borE1 by simple inversions and were found only as heterozygotes. The banding sequence p’borE7 is a result of the translocation described above (Fig. 7C).

**Arm F** has 7 banding sequences (Fig. 6F–K) and was polymorphic in most studied populations. Only the banding sequence p’borF2 was found in hetero- and homozygotic state (Fig. 6F). All inverted banding sequences in this arm, with the exception of p’borF5, are simple paracentric inversions originated from p’borF1. The banding sequence p’borF5 is a complex paracentric inversion that is actually a combination of two inversions that differ banding sequences p’borF2 and p’borF4 from p’borF1 (Fig. 6I).

**Arm G** has 2 banding sequences (Figs 1, 2) and was polymorphic in some populations, mostly from Western Siberia and Far East. The banding sequence p’borG2 is a simple inversion that affects most of the arm, it was found both as hetero- and homozygote (Figs 1, 2B).

Thus, in total banding sequence pool of *Ch.borokensis* now consists of 40 banding sequences.

Beside inversion polymorphism, 5 cases of translocations were observed (Fig. 7). Two were translocations of whole chromosomal arms, involving, in one case, chromosome II (CD) and III (EF), and in the other case – chromosome I (AB)) and III (EF) (Fig. 7A, B, respectively). The translocation presented in Fig. 7C has breakpoints inside chromosomal arms D and E, the resulting banding sequences were described above. Finally, in the population NSK-BR unique larvae was found with two reciprocal whole arm translocations, one involving chromosome I (AB) and II (CD) and the other – chromosomes III (EF) and IV (G) with the arm G joining the arm F and the arm E became a separate chromosome, but unfortunately the slide was damaged before we were able to make photographs.

Rare cases of heterozygosity of the size of centromeric heterochromatin were also observed in populations from Western Siberia, mostly in the chromosome II. B-chromosome was also found in some populations with frequencies up to 30% (Suppl. material 2). In most cases B-chromosome looks like amorphous net of chromatin of various sizes with one or several blobs of heterochromatin (Fig. 2B, D), but in rare cases more regular structures similar to bands can be observed (Fig. 2C). Most often B-chromosome lays in a close association or even form ectopic contacts with centromeric heterochromatin of the arm G (Fig. 2B, C). More rarely it can associate with centromeres of large chromosomes (Fig. 2D) or just lays without visible contacts with other chromosomes, although it is not possible to conclude whether the latter is a true location or just a result of mechanical break of contacts with chromatin of main chromosomes during slide preparation.

For a quantitative analysis of the chromosomal polymorphism, we have used populations with at least 10 specimens (Suppl. materials 2, 3). All populations were checked for deviation from Hardy-Weinberg equation. Only in population RAL-MA a deviation was found in the arm D where number of heterozygotes p’borD1.h’borD2 were higher than expected (P > 0.95).

Overall *Ch.borokensis* can be considered highly polymorphic species. On average 66.1 ± 2.6% of larvae were heterozygous with 1.1 ± 0.6 heterozygotic inversion per larvae. The highest number on banding sequences found in a population from the same date of collection was 18, although for population NSK-BR in total 23 banding sequences were found over all years of observation. On average 12 ± 3 banding sequences were present in a population. Number of genotypic combination of banding sequences varied from 7 to 23 with an average of 15 ± 5. As can be seen from the obtained data, overall populations from Western Siberia show higher level of chromosomal polymorphism than populations from Europe, Eastern Siberia or the Far East.

In five chromosomal arms at least one inversion banding sequence can be considered as alternative (classification according to Gunderina et al. 1999a) as it was present with high frequency at least in some populations and was found in both hetero- and homozygotic state. In the arm A there were two such banding sequences – h’borA2 and p’borA4 – and in arms B, D, F, G alternative banding sequences were h’borB2, h’borD2, p’borF2, and p’borG2. In case of the arm G frequency of p’borG2 reached 100% in many populations from Western Siberia. In arms A and F rare banding sequences (found with low frequencies in several different populations) were also found – p’borA5, p’borA8, p’borA9 and h’borF4. All other banding sequences were unique as they were found only in one larva.

The cytogenetic structure of studied populations was analyzed and populations were assigned types according to Gunderina et al. (1999a). Seven types of cytogenetic structure were found in populations of *Ch.borokensis* (Suppl. material 5), which means that at least some populations differ considerably by frequencies of main and alternative banding sequences. All populations from Europe, Ural and Eastern Siberia belong to type 0 (all main banding sequences are dominant). At the same time most populations from Western Siberia belong to the type G, and some belong to types DG, FG and DFG. Moreover, most populations with G type also show considerably high frequencies of alternative banding sequences h’borD2 and p’borF2, thus we can speculate that alternative banding sequences in arms D, F and G may have some adaptive value in this region. Populations from the Far East had types ADG and F, thus also differ greatly from populations from Europe, Ural and Eastern Siberia. The fact that populations from Eastern Siberia are closer to populations from Europe and Ural than to populations from Western Siberia or the Far East is rather surprising. However, as was suggested in earlier studies, such differences might be due to characteristics of water and detritus in waterbodies from where samples were collected rather than its geographic location, as in chironomids populations from geographically distant regions often show closer cytogenetic structure than populations situated near each other (Shobanov 1994b; Gunderina et al. 1999a, b; Golygina and Kiknadze 2001; Gunderina 2001). To draw more reliable conclusion more populations from different geographic regions is necessary to study along with obtaining data on water qualities.

Cytogenetic distances between studied populations varied from 0.001 (between SVE-OS and SVE-KR) to 0.461 (between KHA-CH and PRI-CH) with the average number 0.155 ± 0.024 (Suppl. material 6). As expected, minimal distances were observed between populations from Europe, Ural and Eastern Siberia, as well as between most populations from Western Siberia, while distances between these two groups of populations and populations from Far East were higher. For the *Ch.plumosus* group average cytogenetic distances between populations of the same species in Palearctic vary between 0.031 and 0.164 (Gunderina et al. 1999b). At the same time the average cytogenetic distance between species calculated on the basis of the data for 7 species is 2.618 ± 0.400 (Gunderina and Kiknadze 2000). The lowest cytogenetic distances were observed between two pare of most closely related species that mainly differ by the size of centromeric heterochromatin – 0.163 for *Ch.agilis*–*Ch.* sp. proper *agilis* (*Ch.agilis*2) and 0.745 for *Ch.borokensis*–*Ch.plumosus*. For other pairs cytogenetic distances varied from 1.142 to 6.669 (Kiknadze 2008). Thus, cytogenetic distances between populations of *Ch.borokensis* are well below the threshold of interspecies values and similar to such values of other species in the *Ch.plumosus* group.

**Figure 8.**
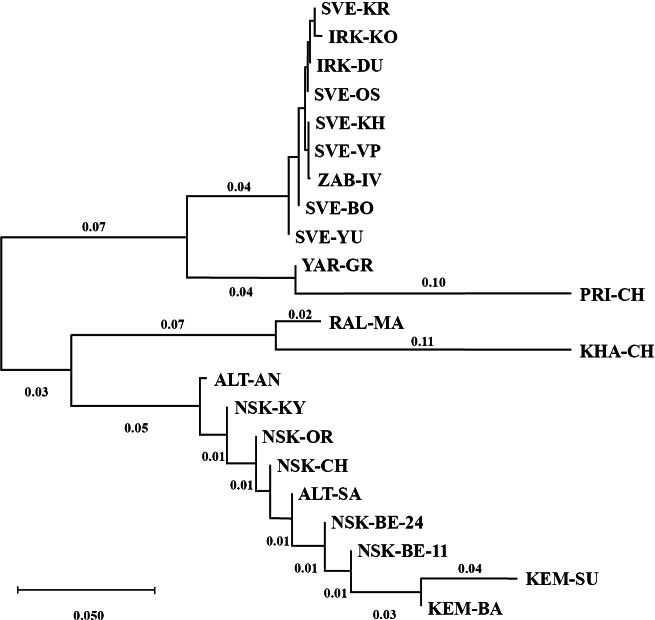
Phylogenetic tree of studied populations of *Ch.borokensis*.

The phylogenetic tree calculated based on the neighbor-joining method is presented on Fig. 8. The clustering of the populations came mostly as expected: populations with closer cytogenetic structure, such as from Western Siberia or from Europe, Ural and Eastern Siberia, fall into same clusters. Still, just as if to prove the point mentioned above, two most distant populations – YAR-GR (Europe) and PRI-CH (the Far East) – form one cluster on the tree. Another cluster of geographically distant populations was formed by populations RAL-MA (Western Siberia) and KHA-CH (the Far East).

Comparison of characteristics of chromosomal polymorphism of *Ch.borokensis* with other studied species from the *Ch.plumosus* group of sibling species, such as *Ch.balatonicus*, *Ch.entis*, *Ch.plumosus*, *Ch.agilis* (Golygina et al. 1996; Butler et al. 1999; Kiknadze et al. 2000; Golygina and Kiknadze 2001; Golygina and Ermolaeva 2021) had shown that it is most similar to the level of polymorphism of *Ch.plumosus* from Palearctic populations, where an average percent of heterozygotes were 63.2% with 0.95 inversion per larvae, and average number of banding sequences in populations was 12.5. Moreover, distribution of inversions between different chromosomal arms are also rather similar. Both *Ch.plumosus* and *Ch.borokensis* have high level of polymorphism in arms A, B, D, and F with highest number of inversions found in the arm A. The difference in polymorphism characteristics between the two species can be found in arms C and G, as in *Ch.plumosus* the arm C is also highly polymorphic and the arm G is completely monomorphic, while in *Ch.borokensis* the arm C is monomorphic in most populations with only 2 unique inversion found and in the arm G frequency of main and alternative banding sequences can vary from 0 to 100%. As both species are very closely related and often live in the same waterbodies, similarity in their polymorphism characteristics is not surprising.

The species range of *Ch.borokensis* spreads from European part of Russia to eastern border of the country (Primorsk and Khabarovsk krai), the most north locality was found in Yakutia near Yakutsk city, and the most south was in Altai krai. Studies of chromosomal polymorphism of other species (Gunderina 2001) had shown that populations from the border of species range often have lower level of chromosomal polymorphism than central populations. Considering the number of inversions and the level of chromosomal polymorphism found in studied populations, it is possible to assume that Western Siberia is the center of the species range and probably its place of origin as populations here have higher level of chromosomal polymorphism and more diverse cytogenetic structures than populations from borders of the species range.

## Supplementary materials

Supplementary material 1 Mapping Data type pdf https://binary.pensoft.net/file/1308261This dataset is made available under the Open Database License (http://opendatacommons.org/licenses/odbl/1.0/). The Open Database License (ODbL) is a license agreement intended to allow users to freely share, modify, and use this Dataset while maintaining this same freedom for others, provided that the original source and author(s) are credited.Veronika V. Golygina, Andrey D. Broshkov, Oksana V. Ermolaeva

Supplementary material 2 Combination frequencies Data type xlsx https://binary.pensoft.net/file/1308262This dataset is made available under the Open Database License (http://opendatacommons.org/licenses/odbl/1.0/). The Open Database License (ODbL) is a license agreement intended to allow users to freely share, modify, and use this Dataset while maintaining this same freedom for others, provided that the original source and author(s) are credited.Veronika V. Golygina, Andrey D. Broshkov, Oksana V. Ermolaeva

Supplementary material 3 Sequence frequencies Data type xlsx https://binary.pensoft.net/file/1308263This dataset is made available under the Open Database License (http://opendatacommons.org/licenses/odbl/1.0/). The Open Database License (ODbL) is a license agreement intended to allow users to freely share, modify, and use this Dataset while maintaining this same freedom for others, provided that the original source and author(s) are credited.Veronika V. Golygina, Andrey D. Broshkov, Oksana V. Ermolaeva

Supplementary material 4 Inversions origin Data type pdf https://binary.pensoft.net/file/1308264This dataset is made available under the Open Database License (http://opendatacommons.org/licenses/odbl/1.0/). The Open Database License (ODbL) is a license agreement intended to allow users to freely share, modify, and use this Dataset while maintaining this same freedom for others, provided that the original source and author(s) are credited.Veronika V. Golygina, Andrey D. Broshkov, Oksana V. Ermolaeva

Supplementary material 5 Cytogenetic structure Data type pdf https://binary.pensoft.net/file/1308265This dataset is made available under the Open Database License (http://opendatacommons.org/licenses/odbl/1.0/). The Open Database License (ODbL) is a license agreement intended to allow users to freely share, modify, and use this Dataset while maintaining this same freedom for others, provided that the original source and author(s) are credited.Veronika V. Golygina, Andrey D. Broshkov, Oksana V. Ermolaeva

Supplementary material 6 Distances Data type xlsx https://binary.pensoft.net/file/1308266This dataset is made available under the Open Database License (http://opendatacommons.org/licenses/odbl/1.0/). The Open Database License (ODbL) is a license agreement intended to allow users to freely share, modify, and use this Dataset while maintaining this same freedom for others, provided that the original source and author(s) are credited.Veronika V. Golygina, Andrey D. Broshkov, Oksana V. Ermolaeva
