# Supplementary material for: ﻿Chromosomal polymorphism in natural populations of Chironomusborokensis Kerkis, Filippova, Shobanov, Gunderina et Kiknadze, 1988 (Diptera, Chironomidae)
Source: Comp Cytogenet. 2025 Apr 15;19:51–74. doi: 10.3897/compcytogen.19.141735 (PMC12015552; doi:10.3897/compcytogen.19.141735)
Supplement: Supplementary material 1 — Mapping [file comparative_cytogenetics-19-051_article-141735__-s001.pdf]

Mapping of banding sequences of *Chiornonomus borokensis*.

Designation of banding sequence	Mapping of banding sequence
p'borA1	1a-2c 10a-12c 3i-2h 4d-9e 2d-g 4c-a 13a-14f 15a-14g 15b-19f C
h'borA2	1a-2c 10a-12a 13ba 4a-c 2g-d 9e-4d 2h-3i 12cb 13c-14f 15a-14g 15b-19f C
p'borA3	no photo presented, not mapped in Keyl-Devai system, suggested mapping in Maximova systed is not detailed enough to identify the banding sequence and convert it into Keyl mapping system (Petrova and Klishko 2005)
p'borA4	1a-2c 10a-12c 3i-2h 4d-8g 13f-a 4a-c 2g-d 9e-a 14a-f 15a-14g 15b-19f C
p'borA5	1a-d 7b-4d 2h-3i 12c-10a 2c-a 1f-k 8b-7c 1e 8c-9e 2d-g 4c-a 13a-14f 15a-14g 15b-19f C
p'borA6	1a-2c 10a-12c 3i-2h 4d-6e 14c-13a 4a-c 2g-d 9e-7a 14d-f 15a-14g 15b-19f C
p'borA7	1a-2c 10a-12c 3i-2h 4d-6a [B15c-B12v] C
p'borA8	1a-d 7b-4d 2h-3i 12c-10a 2c-a 1f-k 8b-7c 1e 8c-g 13f-a 4a-c 2g-d 9e-a 14a-f 15a-14g 15b-19f C
p'borA9	1a-d 7b-4d 2h-3i 12c-10a 2cb 7c-8b 1k-f 2a 1e 8c-9e 2d-g 4c-a 13a-14f 15a-14g 15b-19f C
p'borB1	25s-23f 15g-r 21t-16a 22a-23e 15f-12v C
h'borB2	25s-23f 15g-23e 15f-12v C
p'borB3	25s-23f 15g-r 21t-18a 15ef 23e-22a 16a-17m 15d-12v C
p'borB4	25s-23f 15g-r 21t-18j 22i-a 16a-17e 18g-17f 22j-23e 15f-12v C
p'borB5	25s-n 20a-21t 15r-g 23f-25m 19p-16a 22a-23e 15f-12v C
p'borB6	25s-23f 15g-r 21t-16a 22a-23e 15f-d [A6b-A9e A2d-g A4c-a A13a-A14f A15a-A14g A15b-A19f]C
p'borB7	25s-24k 15l-g 23f-24j 15m-r 21t-16a 22a-23e 15f-12v C
p'borC1	1a-2c 6c-f 7a-d 16a-17a 6hg 11d-12d 4a-6b 11c-8a 15e-13a 3c-2d 17b-22g C
p'borC2	1a-2c 6c-f 8a-11c 6b-4a 12d-11d 6gh 17a-16a 7d-a 15e-13a 3c-2d 17b-22g
p'borC3	Pericentric inversion. Not mapped
p'borD1	1a-3g 11a-13a 10a-8a 18d-a 7g-4a 10e-b 13b-17f 18e-24g C
h'borD2	1a-3g 10b-e 4a-7g 18a-d 8a-10a 13a-11a 13b-17f 18e-24g C
p'borD3	1a-3g 11a-13a 10a-8a 18d-a 7g-5d 21d-18e 17f-13b 10b-e 4a-5c 24g-21e C
p'borD4	Partial translocation with arm E. Not mapped.
p'borD5	Pericentric inversion. Not mapped
h'borE1	1a-3e 5a-10b 4h-3f 10c-13g C (mapped according to Keyl (1962))<br>1a-3a 4c-10b 3e-b 4b-3f 10c-13g C (revised mapping according to Golygina and Kiknadze (2018))
p'borE2	1a-i 3b-e 10b-4c 3a-2a 4b-3f 10c-13g C
p'borE3	1a-3a 4c-8d 10b-8e 3e-b 4b-3f 10c-13g C
p'borE4	1a-3a 4c-5e 9b-6a 9c-10b 3e-b 4b-3f 10c-13g C
p'borE5	1a-i 11b-10c 3f-4b 3b-e 10b-4c 3a-2a 11c-13g C
p'borE6	1a-3a 4c-9a 12g-10c 3f-4b 3b-e 10b-9b 13a-g C
p'borE7	Partial translocation with arm D. Not mapped.
p'borF1	1a-10b 18ed 17d-11a 18a-c 10dc 19a-23f C

p'borF2	1a-10d 18c-a 11a-17d 18d-23f C
p'borF3	1a-10b 18ed 17d-13c 11d-13b 11c-a 18a-c 10dc 19a-23f C
h'borF4	1a-d 6e-1e 7a-10b 18ed 17d-11a 18a-c 10dc 19a-23f C
p'borF5	1a-d 6e-1e 7a-10d 18c-a 11a-17d 18d-23f C
p'borF6	1a-8d 16a-17d 18de 10b-8e 15i-11a 18a-c 10dc 19a-23f C
p'borF7	1a-6c 15f-17d 18de 10b-6d 15e-11a 18a-c 10dc 19a-23f C
p'borG1	Not mapped
p'borG2	Not mapped

Inversions are marked by underline font.
