# Supplementary material for: ﻿Chromosomal polymorphism in natural populations of Chironomusborokensis Kerkis, Filippova, Shobanov, Gunderina et Kiknadze, 1988 (Diptera, Chironomidae)
Source: Comp Cytogenet. 2025 Apr 15;19:51–74. doi: 10.3897/compcytogen.19.141735 (PMC12015552; doi:10.3897/compcytogen.19.141735)
Supplement: Supplementary material 4 — Inversions origin [file comparative_cytogenetics-19-051_article-141735__-s004.pdf]

Origination of inverted banding sequences p’borA5, p’borA8, p’bor A9, p’pluA3, p’pluA7.

p’borA1=p’pluA1 1a-1d-1e-2c 10a-12c 3i-2h 4d-7b-7c-9e 2d-g 4c-a 13a-14f 15a-14g 15b-19f C

hypothetical 1a-d 7b-4d 2h-3i 12c-10a 2c-1f-1e 7c-8b-8c-9e 2d-g 4c-a 13a-14f 15a-14g 15b-19f C

hypothetical 1a-d 7b-4d 2h-3i 12c-10a 2c-1k-1f 8b-7c 1e 8c-9e 2d-g 4c-a 13a-14f 15a-14g 15b-19f C

p’borA5 1a-d 7b-4d 2h-3i 12c-10a 2c-a 1f-k 8b-7c 1e 8c-9e 2d-g 4c-a 13a-14f 15a-14g 15b-19f C

p’borA8 1a-d 7b-4d 2h-3i 12c-10a 2c-a 1f-k 8b-7c 1e 8c-g 13f-a 4a-c 2g-d 9e-a 14a-f 15a-14g 15b-19f C

p’borA5 1a-d 7b-4d 2h-3i 12c-10a 2c-a 1f-k 8b-7c 1e 8c-8g-9a-9e 2d-g 4c-a 13a-13f-14a-14f 15a-14g 15b-19f C

p’borA9 1a-d 7b-4d 2h-3i 12c-10a 2cb 7c-8b 1k-f 2a 1e 8c-9e 2d-g 4c-a 13a-14f 15a-14g

p’pluA3 1a-d 7b-4d 2h-3i 12c-10a 2cb 15a 14f-13a 4a-c 2g-d 9e-8c 1e 7c-8b 1k-f 2a 14i-g 15b-19f C

p’borA5 1a-d 7b-4d 2h-3i 12c-10a 2c-a 1f-k 8b-7c 1e 8c-9e 2d-g 4c-a 13a-14f 15a-14g 15b- -15c-19f C

p’pluA7 1a-d 7b-4d 2h-3i 12c-10a 2c-a 1f-k 8b-7c 15b 14g-15a 14f-13a 4a-c 2g-d 9e-8c 1e 15c-19f C
