# Supplementary figures and images for: ﻿Chromosomal polymorphism in natural populations of Chironomusborokensis Kerkis, Filippova, Shobanov, Gunderina et Kiknadze, 1988 (Diptera, Chironomidae)
Source: Comp Cytogenet. 2025 Apr 15;19:51–74. doi: 10.3897/compcytogen.19.141735 (PMC12015552; doi:10.3897/compcytogen.19.141735)

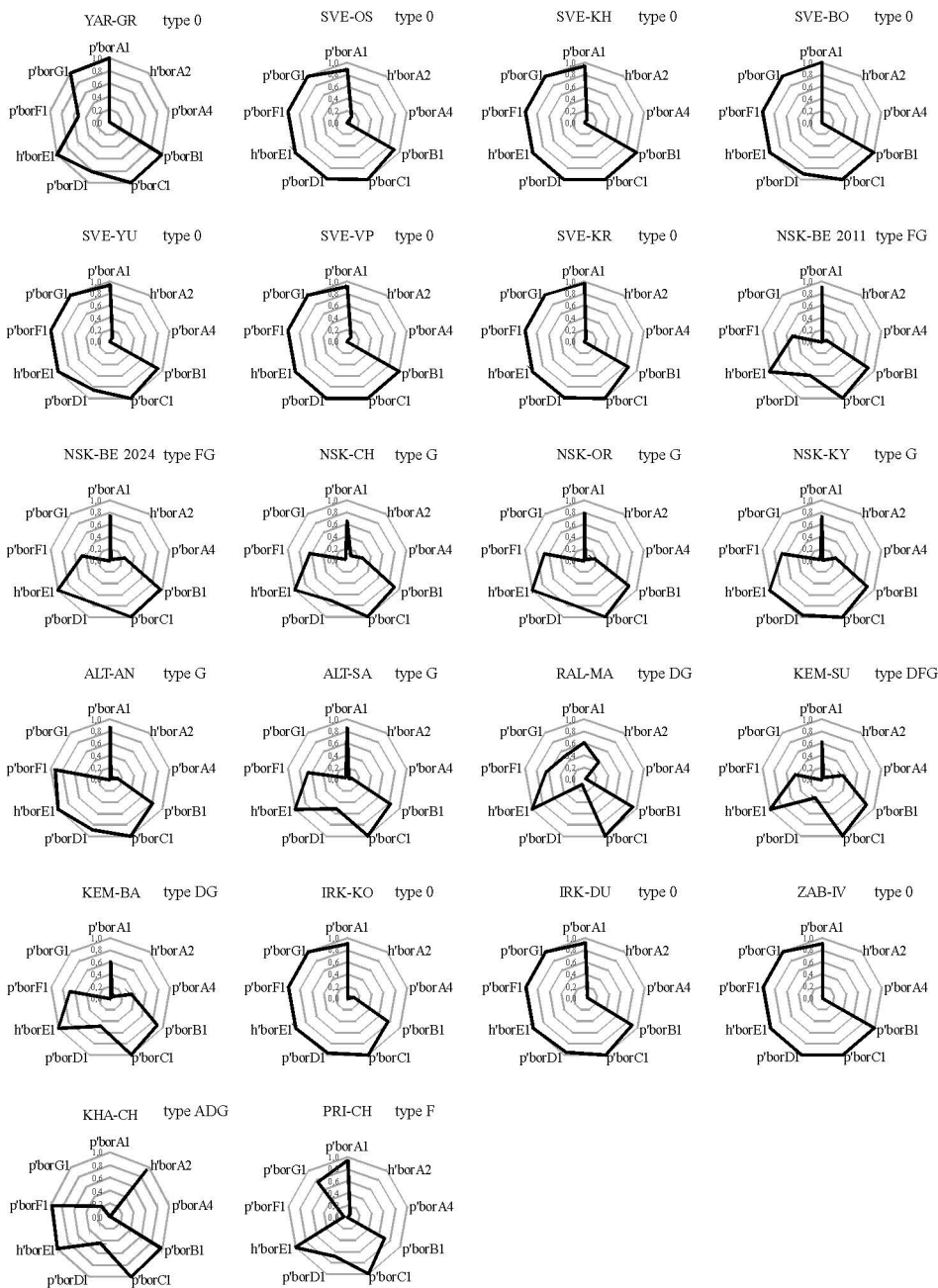

Supplement: Supplementary material 5 — Cytogenetic structure [file comparative_cytogenetics-19-051_article-141735__-s005.pdf]
